# “What I couldn’t do before, I can do now”: Narrations of agentic shifts and psychological growth by young adults reporting discontinuation of self-injury since adolescence

**DOI:** 10.1080/17482631.2021.1986277

**Published:** 2021-10-25

**Authors:** Benjamin Claréus, Tove Lundberg, Daiva Daukantaité

**Affiliations:** Department of Psychology, Lund University, Lund, Sweden

**Keywords:** Narrative analysis, nonsuicidal self-injury, deliberate self-harm, turning points, agency, psychological growth, mental health, well-being, life events, recovery

## Abstract

**Purpose:**

We explore young adults’ narrations of life events in association with nonsuicidal self-injury (NSSI) discontinuation, psychological growth, and agency.

**Methods:**

Transcripts from eleven face-to-face interviews with individuals who quantitatively reported injuring themselves in adolescence (2007–2008) but not in young adulthood (2017) were narratively analysed.

**Results:**

We found that at *starting points*, a period preceding an agentic shift in the narrative, participants endured stressful living conditions and mental illness. During this period, participants perceived *no point* in trying to initiate change because they did not perceive themselves as having the capacity to do so and nor could they adequately utilize any formal or informal support. After a *turning point* that enabled agency due to gaining a sense of belongingness, liberation, or perspective, participants underwent a process of attaining psychological well-being. However, narrating psychological growth also required *momentum points*, encompassing the management of and moving on from stressful contexts, along with the recognition of milestones marking improvement relative to the *starting points*.

**Conclusions:**

NSSI discontinuation was narrated in conjunction with psychological growth when participants also experienced themselves as situated within an agentic context, because agency is understood as necessary to react to and manage current and future life circumstances.

## Introduction

Self-injurious behaviours such as cutting or hitting oneself are relatively prevalent in adolescent populations (Swannell et al., 2014) but less so among young adults (Daukantaité et al., 2020; Moran et al., 2012), suggesting that most adolescents who injure themselves discontinue before reaching adulthood. Discontinuation of self-injury is usually treated as a positive sign reflective of improved mental health and psychological well-being. Nevertheless, repeated self-injury in adolescence indicates not only that the psychosocial conditions of the individual are currently poor (Ammerman et al., 2017) but also that these conditions might affect them negatively into adulthood (Borschmann et al., 2017; Daukantaité et al., 2020; Mars et al., 2014). Discontinuing self-injury is therefore not a sufficient indicator of well-being in young adults (Shaw, 2006). However, understanding when discontinuation is reflective of greater well-being might provide important insights into how vulnerable adolescents diverge from an adverse trajectory onto a path towards psychological health.

Earlier work suggests that improved living conditions play an important part in discontinuing self-injury (Buser et al., 2014; Shaw, 2006; Sinclair & Green, 2005; Whitlock et al., 2015), although certain life events may be salient to some individuals but not others (Rissanen et al., 2013). Therefore, the current study explores the life conditions that young adults associate with the discontinuation of self-injury. The study focuses on narratives spanning from adolescence (aged 14–15 years) to young adulthood (aged 25 years) in a sample from a Swedish cohort who reported having injured themselves in a quantitative survey in adolescence (2007, 2008) but not in adulthood (2017).

In the following sections, we define self-injury within the context of the current study, after which we present current research on discontinuation of self-injury and how it relates to psychological well-being. We also introduce the theoretical concept of agency, which is used in the study to delineate the contexts in which individuals narrate themselves as being able to flourish and the contexts that they do not.

### Nonsuicidal self-injury

There are multiple conceptualizations of self-injury utilized in research and clinical practice (McAllister, 2003). For the current study, we utilize Nock’s (2010) conceptualization of nonsuicidal self-injury (NSSI) as an umbrella term for behaviours that cause direct physical harm to one’s body, without an intent to kill oneself. This definition includes behaviours such as cutting, burning, hitting, or biting oneself (Gratz, 2001), but excludes instances where bodily harm is indirect (e.g., injuring oneself by sex or substance use; Nock, 2010).

Although self-injurious behaviours are more common in clinical populations (Gandhi et al., 2018), Brunner et al. (2014) estimated a 27.6% lifetime prevalence of self-injury among European citizens overall, with 7.8% of the population reporting to have injured themselves more than four times in their lives. Swannell et al. (2014) reported that the prevalence is higher among adolescents (10–17 years) than among young adults (18–24 years) and adults (≥25 years), and Gandhi et al. (2018) suggest that the first self-injuries tend to occur around 14–15 years old. Adolescents who have self-injured sometime in their life often express thoughts about injuring themselves again (Kelada et al., 2018; Wadman et al., 2017), while 20% of adolescents who have never self-injured themselves report self-injury ideations (Berger et al., 2017; Gillen et al., 2019). Reported reasons for repetitive engagement in NSSI include using self-injury to manage painful emotional states when lacking alternative resources (Edmondson et al., 2016), or as a response to social stressors in combination with low personal control (Brown & Kimball, 2013; Ekman & Söderberg, 2009). In both instances, repeated NSSI might signal that a particular adolescent is not only currently in significant distress (Ammerman et al., 2017), but is situated in an intra- and interpersonal context that can negatively affect their mental health and social development in the long term. For example, reporting NSSI in adolescence is associated with lower psychological well-being and more pronounced psychopathology (Daukantaité et al., 2020) as well as lower socioeconomic status including lower income, lower educational attainment, and a greater likelihood of unemployment in adulthood (Borschmann et al., 2017; Mars et al., 2014), as compared to not reporting NSSI.

### Discontinuing NSSI

While thoughts about or engagement in NSSI are relatively common among adolescents, most do not injure themselves repeatedly in adulthood (Daukantaité et al., 2020; Moran et al., 2012). In previous research, reduced or discontinued NSSI engagement has been understood as a conscious and gradual intrapersonal recovery process with interacting cognitive, behavioural, and relational aspects (Buser et al., 2014; Kruzan & Whitlock, 2019). For example, qualitative studies show that a desire for change (e.g., Gelinas & Wright, 2013; Ryan-Vig et al., 2019) or a deliberate decision to stop (e.g., Hamza & Willoughby, 2014; Kelada et al., 2018; Lindgren et al., 2018; Long et al., 2015; Rissanen et al., 2009) are instrumental to initiating the discontinuation process. The desire or decision often follows after a personal realization that NSSI negatively affects one’s relationships (Buser et al., 2014; Gelinas & Wright, 2013; Shaw, 2006), is futile or meaningless (Gelinas & Wright, 2013; Holliday et al., 2018; Rissanen et al., 2013), or could interfere with current life and prospects (Shaw, 2006; Weber, 2002).

Upon deciding to discontinue NSSI, an individual may then actively engage in various strategies to reduce their engagement in self-injurious behaviours. Such strategies may include engaging in behaviours that distract oneself from NSSI ideations (e.g., listening to music, exercising, smoking; Kool et al., 2009; Rissanen et al., 2009; Ryan-Vig et al., 2019; Tofthagen et al., 2017; Wadman et al., 2018) or reappraising affects and thoughts that preceded previous self-injurious episodes (Andrews et al., 2013; Horgan & Martin, 2016; Kruzan & Whitlock, 2019). One may also utilize interpersonal strategies, such as seeking emotional support from family, friends, or healthcare professionals (Kool et al., 2009; Tofthagen et al., 2017). A recent literature review found that, among those who use relational resources to discontinue NSSI, informal networks including friends and family are generally preferred over formal sources such as healthcare or social workers (Simone & Hamza, 2020). Yet, even among individuals who sought formal support, some found clinical interventions unhelpful or of little significance in the actual process of discontinuing NSSI (Kelada et al., 2018; Rissanen et al., 2013; Whitlock et al., 2015), whereas others found it actively hurtful or damaging to their mental health (Long, 2018). However, while NSSI discontinuation can be conceptualized as a salient stepwise process of recovery for some (e.g., Lewis et al., 2019; Tofthagen et al., 2017), for others it is more the result of self-injury no longer being useful. Indeed, someone who self-injures might not perceive the behaviour as either *a* problem or *the* problem (Long et al., 2015; Shaw, 2006), constructing it as an reasonable and effective way to cope with current and future hardships (Kelada et al., 2018). Qualitative research has shown that, even for individuals who have repeatedly and severely injured themselves, NSSI discontinuation can be attributed to a change in living conditions (Buser et al., 2014; Shaw, 2006; Sinclair & Green, 2005; Whitlock et al., 2015) and subsequent life improvements (Rissanen et al., 2013), rather than being located within a discourse of recovery. Therefore, discontinuation of NSSI is made possible by improved life circumstances separate to or in conjunction with the implementation of different cognitive, behavioural, and relational strategies that diminish or remove the “urge” or “desire” to self-injure (Kelada et al., 2018; Kruzan & Whitlock, 2019; Lewis et al., 2019).

### Agency and psychological growth

As expected from the positive psychological and social change processes associated with NSSI discontinuation, quantitative research shows that discontinuing NSSI, compared with continuing it, is associated with higher well-being and improved mental health over one- (Hamza & Willoughby, 2014; Kiekens et al., 2018) and three-year periods (Kiekens et al., 2017). However, discontinuing NSSI does not equal psychological recovery or growth *per se* (Lewis & Hasking, 2021). For example, Shaw (2006) argues in her study of six adult women that “[…] stopping self-injury, in itself, is not a reliable indicator of greater psychological health unless it is accompanied by shifts in other domains of a woman’s life” (p. 171). Beyond addressing potential concurrent mental health concerns (e.g., Lewis et al., 2019), such shifts include contextual changes such as making life commitments and forging relational ties that longitudinally facilitate positive psychological development (Shaw, 2006). This process could partly include building resilience (Lewis & Hasking, 2021) and agency. Whereas resilience describes the active process of reframing past adversities and coping with current adversities (Luthar et al., 2000), agency, as defined by Bandura (2006), is the broader process of constructing oneself as someone who can act on their current life circumstances. Agentic acts are directed towards a visualized future while also potentially, but not necessarily, coping with the contextual influences pulling that person in another direction. Agency encompasses concepts of mastery, self-actualization, and autonomy (Bandura, 2006), making it an integral part of eudaimonic well-being. Eudaimonia is typically defined as the pursuit of happiness and flourishing beyond the absence of destructive behaviours, mental illness, or detrimental living conditions (Ryff & Singer, 2003), and it is important for constructing oneself as thriving and living a meaningful life as opposed to just getting by with one’s current resources.

Psychological growth from the perspective of eudaimonic well-being can be narrated temporally as making successive progress by integrating various experiences and events with the self through the larger themes of agency, communion, and learning (Bauer & McAdams, 2004). Building on these theories, situating self-injury within one’s agentic capacity might be helpful for understanding when NSSI discontinuation is indicative of psychological growth; from an NSSI recovery framework, it would be helpful in understanding when one can realize their strengths, address different adversities, and feel a strong self of self-efficacy (Lewis & Hasking, 2021). This exploration must also consider context, as an individual’s efficacy can be both limited and reinforced by current life circumstances, such as social structures and fortuitous events (Evans, 2007). Earlier work has found that life circumstances perceived to be restricting agency, such as institutionalization or foster care relocation, are associated with NSSI engagement (Donskoy & Stevens, 2013; Wadman et al., 2018); these findings are sometimes explained as that cutting or other methods of self-injury can reinstate a sense of control in otherwise chaotic life situations (Csordas & Jenkins, 2018). Moreover, a content analysis by Rissanen et al. (2013) showed that changing social networks and life events (e.g., starting therapy, finding a significant other or confidant) are important factors in ceasing self-cutting. Besides altered circumstances and proactive choice, we must also consider how psychological growth is nurtured or sustained in the long run (Gilligan, 2009), such as the momentum some individuals experience when ceasing self-harm and starting to feel better generally (Shaw, 2006).

### The current study

There is considerable research on NSSI discontinuation, which has highlighted the positive intra- and interpersonal changes that accompany discontinuation and that simultaneously improve mental health and well-being. This psychological growth process, however, is not directly attributed to the cessation of NSSI itself, but can occur via diverse external events that alter the individual’s living conditions and psychological health. Further understanding of how life events can change an individual’s psychosocial context can give us important insights into not only how some external events facilitate NSSI discontinuation and recovery, but also how adolescents diverge from the negative developmental trajectories implied by their previous life conditions and mental health issues. Because agency encompasses an individual’s perception of whether they can change their current context, and is associated with our current definition of well-being, it could also help explain when and how NSSI discontinuation or particular life events are associated with psychological growth and when they are not. Accordingly, our research questions were: “*How do young adults narrate life conditions and events before, during, and after NSSI discontinuation,”* and, *“how do young adults construct their agency in relation to the life events that shaped their understanding of whom they are today?”*

## Materials and methods

### Participants and recruitment

The 11 participants included in this study were recruited via a longitudinal project examining NSSI, emotion regulation, and interpersonal relations in a Swedish community sample of adolescents followed-up after 10 years (in young adulthood). The original sample included all 7th and 8th grade students within all public schools in a Swedish municipality. Quantitative data were collected in 2007 (T1: N = 991, M_age_ [SD] = 13.7 [0.68]), 2008 (T2: N = 984, M_age_ [SD] = 14.8 [0.69]), and 2017 (T3: N = 557, M_age_ [SD] = 25.3 [0.68]; cf. Daukantaité et al., 2020). Of the 557 young adults who completed the questionnaire in 2017, 228 indicated an interest in participating in a follow-up interview.

Our main aim with this follow-up qualitative portion of the project was to obtain a more holistic and experience-based view of mental health and well-being in young adulthood. This included aspects not covered by the quantitative portion, particularly participants’ own view of their life course and what had affected them the most during their upbringing. We aimed to interview about 30 individuals, prioritizing those who (relative to the rest of the sample) scored very high or very low on a composite index of mental health. This index comprised both positive and negative aspects of mental health reported in adolescence and young adulthood, including self-injury, psychological distress, life satisfaction, and flourishing. Additionally, since the local ethical review board required that all interviews be conducted face-to-face, we considered a 2-hour travel time (one-way) from Lund University as viable (travel costs were covered by project funds). In total, we invited 119 individuals to participate. Twenty-eight individuals agreed to participate, 55 individuals declined, and 36 could not be reached by phone/mail. Two-tailed Welch’s *t*-tests could not statistically discern a difference between those who were invited but did not participate (n = 91) and those who were interviewed (n = 28) on T1/T2 measures of NSSI, psychological problems, and depressive symptoms (*p* = 0.22‒0.87) or T3 measures of NSSI, life satisfaction, flourishing, resilience, depression, anxiety, stress, and emotion dysregulation (*p* = 0.10‒0.49).

Because the quantitative portion of the longitudinal project involved exploring the associations and predictors of NSSI, the interview protocol included questions about participants’ prior experiences of self-injury as well as questions about their mental health and life situation. The self-injury questions were initially approached with the aim of assessing the intentionality of NSSI in participants’ life narratives. However, after an initial reading, we found that no participant recounted any lived experiences of NSSI as particularly salient to their life course; rather, NSSI was one of many coping strategies utilized for managing psychological distress. Additionally, most participants who recounted a lived experience of NSSI in adolescence described themselves as currently doing better than before. As the capacity for action was a recurrent theme in the interviews, we ultimately formulated the research questions that guided the theoretical framework and the analysis reported in the current paper.

These research questions guided us to select the 11 interviewees whose quantitative data indicated that they had discontinued repetitive NSSI between adolescence and young adulthood—that is, who reported more than five instances of NSSI at T1/T2 but not at T3 as assessed by the Revised Deliberate Self-harm Inventory (DSHI-9 r, Gratz, 2001; Lundh et al., 2011). Five of these participants identified as women and six as men, and they were all aged 25‒26 years. As our definition of repetitive NSSI did not account for the potential relevance of someone who reported fewer instances of NSSI or even none at all, the remaining interviews were also checked for narrations of NSSI discontinuation/recovery or similar. This process revealed no additional cases.

### Procedure

The interviews were conducted in 2018 at Lund University by a licenced clinical psychologist with clinical and research experience of NSSI and mental health. Participation was reimbursed with lottery or cinema tickets, as well as monetary compensation for travel expenses. All interviews followed a semi-structured interview guide that outlined the general themes (e.g., “Do you remember the questionnaires that you filled out in 2006 and 2007? What can you remember about that time?” and “What would you say has affected your life the most?”) and included some prompts (e.g., “How would you describe your family situation when you went to lower secondary school?” and “Has something difficult happened in your life that you would like to talk about?”). To allow the participant to talk about what was most important to them, these prompts included clarifications or asked them to expand on specific topics and narratives already mentioned (Clausen, 1998).

Because the invitation letter was directed to all individuals in the qualitative portion of this project, neither it nor the information letter mentioned NSSI specifically. Instead, it explicated that the interview theme was their life story from lower secondary school onwards, including but not limited to distressing life experiences that had influenced them. Mentioning NSSI explicitly could have constructed some participants as having an experience of particular research interest, thereby the interview on that topic and potentially diverting attention from other episodes that the participant considered equally or more salient aspects of their life course (Potter & Hepburn, 2005). We wanted to avoid this situation because previous research has shown that not all individuals who have previously self-injured consider it salient of their lived experience (Kelada et al., 2018; Long et al., 2015). Moreover, the interview study as a whole did not have an explicit focus on self-injury. Hence, *all* interviewees were prompted about NSSI in a context-sensitive and considerate manner (e.g., “Did you try to harm yourself in any other way than what you said about [intentionally starting] fights?” or “One thing also mentioned in the questionnaires was self-harm, or things you do that might not be good for you. How was that for you [when growing up]?”), but the interview did not linger on the theme if the participant denied having injured themselves or framed any recalled events as unimportant to their story. Moreover, if the participant—either verbally or through their mannerisms—expressed discomfort at this question or any other potentially sensitive or stigmatized topic that arose spontaneously or through the interviewers’ questions, the interviewer reiterated the participant’s right to deny answering questions and moved on.

### Data analysis

The data were analysed using a narrative analysis inspired by the analytic steps described by Crossley (2000) and Hiles and Cermák (2008). The interviews were first transcribed verbatim in NVivo 12 for Windows (QSR International). The first author read the transcripts several times to familiarize himself with the narratives, and following Crossley (2000), coded his initial impressions in NVivo about the tone (e.g., optimism, pessimism), imagery (e.g., word usage, identity constructions, descriptions), and broader themes and patterns in nodes. These nodes were then utilized to delineate segments in a full read-through of the transcripts—that is, self-contained narrative episodes about a participant’s internal and external contexts (Hiles & Cermák, 2008). Utilizing NVivo’s mapping function, these segments were arranged in an event-history matrix attempting to capture the approximate temporal order of events (Miles et al., 2014). Any connected successions or relations beyond this temporal order were added as well, such as intentionality or thematic coherence (Mishler, 1995).

Upon reviewing the event-history matrices of each transcript, we noted considerable agentic shifts in the narratives occurring at several *turning points*. By turning points, we refer to particular events that participants perceived to significantly alter the conditions of their everyday lives (Hareven & Masaoka, 1988). Narrations of turning points influence and are influenced by psychosocial processes occurring before and after the actual events (Clausen, 1998; Pickles & Rutter, 1994). So in addition to turning points, we developed our framework to include *starting points, no points*, and *momentum points* to better understand why some events, but not others, contributed to agentic changes in the life situation. In line with Wheaton and Gotlib’s (1997) reasoning that turning points are only recognized as such against a baseline, *starting points* were included as descriptions of the participants’ psychosocial context before a particular turning point. We also directed attention to *no points* within the descriptions of *starting points. No points* were life events that participants perceived as having the potential to become turning points, but that did not occur or were associated with no, or only temporary, change. *Momentum points* were events that occurred after a turning point that affected the extent, continuity, and duration of the change associated with the turning point (cf. Gilligan, 2009; Hareven & Masaoka, 1988).

After creating nodes representing each kind of point, participants’ event-history matrices were utilized to code individual segments as instances of a particular point. Throughout our reading of each new node, crosstabs and matrix coding was used to assist in finding commonalities and dissimilarities between different kinds of events and their effects. During this process, we noted that one of the participants (Pa11) did not narrate a clear turning point or any clear momentum points, but she did mention several no points. Hence, we retained Pa11’s data as separate for comparison purposes when aggregating the results. Before the analysis was finalized, all quotes were translated from Swedish to English.

### Ethical considerations

The regional ethical review board in Lund approved the quantitative (2006/49; 2016/1059) and qualitative (2018/537) portions of the study. Before the interview was booked, participants received written information about the study and their rights to discontinue participation, and confidentiality. Before the interview, the interviewer verbally went through the information letter, answering any questions the interviewee might have before they provided written consent to have their interviews recorded, transcribed, and analysed.

Interviews were held in a remote office where the participants would not run into any university employees or students when arriving at and leaving the interview. At the end of the interview, participants were asked to reflect on their experience of taking part in the study, in order to confirm their emotional state. The interviewer asked questions about their mood and the participants filled in a visual analogue scale, where they indicated their mood by ticking a line ranging from 0 cm = *in a very negative mood* to 10 cm = *in a very positive mood*; a ruler was used to determine their score (Biddle et al., 2013). Participants completed the scale before and after the interview, and submitted it to a postbox emptied by another research group member so that the interviewer did not see their response. A Wilcoxson test showed a slight but non-significant improvement in participants’ mood from before (*Md *= 84.25) to after the interview (*Md *= 93.7; *z* = −0.77, *p* = 0.44). When asked, none of the participants suggested that the interview included topics they did not anticipate or expressed explicit concern about the topic of NSSI or destructive behaviours in particular. A majority of participants indicated that their participation was interesting and beneficial for understanding themselves, or mentioned that sharing negative past experiences was a meaningful way of helping others.

After the interview, participants were provided with written contact information for the research group so that they could indicate any further concerns or ask questions about their experience, data, or anything else. All participants who requested access to their quantitative data from 2007 and 2008 were given copies of their surveys.

## Results

In recounting their past experiences, participants situated their *starting point* within a period of psychosocial adversity. Because this adversity was constructed as inescapable and unsolvable, participants described how they could only endure it. While the *turning points* differed between participants and did not change the external conditions for everyone, they did enable a sense of agency. At subsequent *momentum points*, participants utilized that agency to construct narratives around managing adversity and moving on. Moreover, by recognizing their ability to bring about positive change, participants acted towards sustaining and initiating change within domains that contributed to their psychological well-being and mental health. Consequently, participants were able to construct narratives of growth after the *turning point* not seen as possible at the *starting point*. An overview of these themes is available in Table I. Below we present our analysis of the *starting, turning*, and *momentum points* on a between-individual level, highlighting the commonalities in the sequencing even when the events were different.

**Table I.** Overview of the narrative themes and subthemes

Narrative theme	Definition	Subtheme	Subtheme description
Starting points	Participants’ psychosocial context before the turning point	Before, I just endured	Narrations of stressful life events (e.g., bullying) or mental illness (e.g., depression) that could not be adequately dealt with, only endured (by e.g., NSSI)
		Before, there was no point	Imagined or actual attempts to change one’s context (e.g., by seeking social support) that ended up not occurring or were associated with no, or only temporary, change
Turning points	An event that enabled a sense of agency within a domain important to the participant	Then, I belonged	Sensing a belongingness with other people who entered their lives meant that participants could take on current challenges without apprehending failure
		Then, I was liberated	After being liberated (e.g., through physical separation) from the starting point context, participants could start prioritizing their own wishes and needs
		Then, I gained perspective	Participants suddenly realized how self-realization was possible and which choices would be detrimental thereof
Momentum points	Events that affected the extent, continuity, and duration of the turning point, increasing perceived well-being over time	After, I will manage	Participants found it possible and worthwhile to try to proactively manage adversity, either by themselves or by seeking social support
		After, I can move on	If adversity could not be managed, this could be reconstructed into learning experiences about oneself or about how to help others
		After, I bring about change	Life transitions were understood as achievements that signified growth, which affirmed participants’ beliefs that they could bring about change

### Starting points

Before the *turning point*, the participants recounted stressful experiences such as bullying (Pa2, Pa9, Pa10), observations of drug abuse or mental illness in caretakers (Pa3, Pa7, Pa10), conflicts with significant others (Pa1, Pa8), homelessness or unemployment (Pa5, Pa6, Pa8), and overwhelming school-related or occupational demands (Pa1, Pa4). Participants also narrated various mental health issues, sometimes in psychiatric nomenclature such as “I had my depression” (Pa4) or “[I] had a lot of anxiety” (Pa6), but also as being “afraid” (Pa2, Pa7, Pa9, Pa10), “sad” (Pa3, Pa8, Pa9, Pa10), “tired” (Pa1, Pa4, Pa6, Pa8), or “lonely” (Pa2, Pa3, Pa7). Some retrospectively perceived their contextual stressors as causing their mental health issues (e.g., Pa9: “[The bullying] was weighing you down back then”) or that the relationship was reciprocal (e.g., Pa4: “[I] had a very hectic lifestyle [and] I started to feel bad, and then you enter into a vicious circle and it gets worse and worse”). Because participants believed that there was *no point* in trying to resolve the situation, they used various cognitive and behavioural strategies to endure life.

#### Before, I just endured

At their *starting point*, participants described how their agency to change their situation was limited, narrating their past living conditions as inevitable, unresolvable, or outside their control. For the participant who had not experienced a turning point, this extended towards the future as well: “The future will nevertheless remain like it is right now” (Pa11). Such constructions were associated with memories of suicidal thoughts or attempts in five participants. For example, Pa2 said, “It was a bit like this for me, that I was thinking about if I would, if living was worth it or how it was”. This participant (Pa2) continued describing how her past life “was a little bit about surviving”. Participants who did not recall suicidal ideations also narrated a sense of enduring life on a day-to-day basis: “A lot about taking one step at a time […] Did the minimum required for being able to live” (Pa8). Enduring, or just surviving, was possible through various avoidant coping strategies that were used interchangeably depending on what was considered functional and available. The most common strategy was avoiding emotional processing of a stressor via cognitive and emotional detachment (e.g., Pa11: “I would rather […] shut myself off”), or distraction: ”You were in your own bubble, with all your fantasies” (Pa9). Participants also said that they modulated emotional reactions to unavoidable stressors into something more manageable by injuring themselves (e.g., Pa7: ”But [cutting] became like an expression for, you got to express it, like relieving the pressure”) and being aggressive: “I do not need to confront the problems, [I: Mm] but it, it turns into anger instead” (Pa6). Although participants noticed that this could have negative consequences, they did not consider it a problem insofar that they could not manage otherwise: “[Cutting was] nothing that I did daily or anything like that, but it was like, only when it was at its worst. […] I did not think of it as a problem” (Pa8). During this part of the narration, discontinuation of self-injury was constructed as choosing to utilize other strategies for endurance, rather than as a sign of improved well-being. Pa2 said, for example, “[self-injury] did not work out for me like I had thought it would.” Doing something other than enduring would have required their situation to change, but as elaborated on below, this was constructed as impossible at the *starting point*.

#### Before, there was no point

At the *starting point*, participants acknowledged that they would be reliant on the help of others to change the situation or just to feel better. One participant said that confiding in others was described to them as a good thing: “People said that, you know, talking and such things do help, [I: Mm] that was what you were told” (Pa10). However, despite acknowledging that they should and could have reached out to others such as relatives, friends, teachers, or health professionals, participants also described themselves as hesitant to do so. Hesitation was grounded in beliefs that outside intervention might inadvertently worsen their circumstances (e.g., escalating friction between caretakers); that the consequences would go against their wishes (e.g., a social worker separating one from one’s family); or that nobody they trusted had the authority to initiate change (e.g., only trusting a friend who could not intervene in their caretakers’ actions). Rather than risking such negative consequences, some participants described keeping up a façade: “Many adults […] saw this façade that I basically was, but didn’t understand what was actually going on in my life” (Pa1). If others understood their situation and offered help, some participants such as Pa10 decided to lie or to keep quiet: “Mum and dad [wanted] me to visit a psychologist […]. Then they forced me to go [to the psychologist], but I never told them anything.” While Pa2 admitted that she was suffering through daily ostracism and physical violence at school when her mother unwittingly found out about it, it did not influence her situation:

Pa2: My mother helped me and talked [with me], and there was a lot of communication with the school and the teachers. But, well, I don’t know. It sort of helped that someone was on my side, but it didn’t fix the situation, it was the way it was.

The eight participants who said that they had been in contact with the local social services, child and youth psychiatric services, or adult psychiatry units agreed with Pa2 that social support helped only temporarily, if at all. Pa8, for example, regularly visited a psychotherapist but said that it did not improve things in the long run: “It was all just a bit of talking about it, and such, [and] you feel better for 15 minutes, 30 minutes. Then it is like back to normal again. [I: Mm] There was nothing more to it.” Others explained how at the time they were unable to engage in therapy because they were too occupied with the situation (e.g., Pa4: “In periods I was too dep- well, too unwell to internalize anything”) or the counsellor diverted attention from what they wanted to talk about to other topics: “We always spoke about, how are things today and so. [I: Mm] When I actually wanted to talk and work through everything that had been” (Pa3). As participants experienced informal or formal supports as not extending beyond the moment or as inadequate for their situation, these episodes could be interpreted as *no points*. Moreover, the perceived lack of agency in their context was paramount to participants’ perceiving them as *no points*, as participants could not utilize the available resources or benefit from the tools provided to them.

### Turning points

Most interviewees recounted an event that significantly contributed to altering the conditions they endured at the *starting point*. Some stated that the *turning point* was imminently “life-changing” (Pa1), while others retrospectively acknowledged how it impacted their previous situation. The characteristics of *turning point* events differed between participants, but all these events enabled agency. Agency was important for narrating the initiation of psychological growth beyond the perceived restrictions of their *starting point*. However, the participant who did not narrate a turning point continuously situated her well-being as contingent on factors outside her control, such as fate:

Pa11: I believe or think that it, it was meant to be. Everything.

I: Mm, okay, things have turned out as good, as good as they can be, or so?

Pa11: Yeah, I believe so.

That same participant (Pa11) also noted that if she had made different choices in life, a particular event might have affected most aspects of her life, albeit in an unknown way: “That was the moment when I decided […] about how everything would become.” Therefore, enabling agency meant not only that participants chose to make a change based on the resources the turning point had made available, but also acknowledge that the outcome of that change would be difficult to anticipate. How participants who narrated a *turning point* described this enablement and its effects on their immediate living conditions were grouped into three subthemes: *Then, I belonged; Then, I was liberated;* and *Then, I gained perspective*.

#### Then, I belonged

Pa5 and Pa8 associated their *turning point* with gaining a sense of belongingness, as both met someone who either invited them into a community or supported them. For example, Pa5 started a new job after applying to “all jobs that existed,” and expressed that the belongingness he felt there meant a lot to him at that time: “Then I joined a team, that had worked together for a long time. […] That meant a lot to me, [I: Mm] It did. [I: Mm] I entered a community”. While Pa5 brought about this event by actively looking for a job, he did not expect it to become a *turning point* in his life through connecting with people at his workplace. Pa8’s experience was similar. He said that he had always had to “manage on my own, more or less,” but at the time a newfound partner supported him through an episode of homelessness, which was something he had not anticipated happening:

Pa8: I told [him] at the beginning, like, we can meet, we can hang out, and such. But like, right now I did not want a relationship, because I was very deep down. But it is thanks to him that I, like, he was there for me all the time [I: Mm] and supported me.

Later in the interview, Pa8 said he “started building from there” by trying to find a permanent home and job despite previous successive setbacks, recognizing his agency to do so without apprehending failure. Agency waas hence enabled by perceiving that one had a secure foundation of unconditional belongingness, such as by someone who was “there for me all the time” (Pa8) or feeling “at home” (Pa5), which negated any potential risks.

#### Then, I was liberated

Other participants also described that their *turning point* liberated them from an inhibiting context, allowing them to be or act in accordance with their values or wishes. Pa2, Pa9, and Pa10 all had experienced bullying before changing schools. In their new contexts, they expressed a sense of freedom as in “you could do everything” (Pa9) or in terms of “you were given a new chance” (Pa10). One participant (Pa2) further highlighted the unexpectedness of the opportunity, claiming to have been “completely shocked” that her new peers wanted to talk to her and impartially welcomed her participation during class activities. Therefore, acting in accordance with one’s values and wishes became possible upon being physically separated from a bullying situation. Pa3 and Pa7 both described how moving meant that they could avoid people that negatively affected them:

Pa3: Back then, well, I didn’t want to visit my mother and I had been telling people that for so many years, and now it was such a relief, that now I don’t need to visit my mother because I get to decide that for myself. [I: Mm] I didn’t want my stepmother in my life because she is just destructive and made me feel bad, it was such a relief back then that now I don’t need to see her anymore.

Similar to Pa3, other participants (e.g., Pa6) expressed a desire to act, be, or feel differently before their actual physical separation from the *starting point* context. Pa6 tried for many years to improve his mental health by various means, but it was not until he recently tried a new medication that his outlook changed. He explained: “It felt like the first time that I […] became who I should be. So like, what I couldn’t do before, I can do now.” Therefore, while the participant desired change before the *turning point*, the *turning point* itself affected their context such that change became possible from a long-term perspective.

#### Then, I gained perspective

In addition to events that helped participants feel that they belonged or were liberated, others described events that caused a shift in their way of thinking. This was the case for the two participants whose *starting points* were being overwhelmed by work- and school-related obligations. Pa1, for example, described how he felt that he had had enough of his old lifestyle of working around the clock and decided to go abroad. There he met someone who showed him an alternative way of being:

Pa1: Then I felt like, no, I just need to get [away from home] if only for a month. So I took the money that I had and visited [a country], and I spent a month there and I met possibly the most, really, wonderful human that exists on this planet, and he inspires me so much that afterwards, I felt like, this is the kind of human I want to be.

Pa1 further described how this contextual change was essential for him to start re-evaluating his life, as he previously “was unable to think in that way” about himself and his life. He expressed surprise that his trip had turned out to be a transformative experience rather than just a temporary escape. Pa4’s experience was similar. She described how burnout resulted in her gaining, for the first time, distance from the prestigious educational context she had been situated in since elementary school. This event helped her understand that she wanted something different in her life:

Pa4: To get away from, eh, well, the stress from studying and trying to find yourself. Eh. Simply put, getting a perspective on things. […] I really needed that time to, eh, get a slightly, well, different perspective and get like other values, really.

Pa4 described how gaining perspective on her life made her work towards self-realization, such as spending less time on homework and more time on reinvigorating hobbies. Similarly, Pa1 said that “since then I have tried to reach personal development,” highlighting that what the *turning point* had enabled were actions that aligned with the participants’ newfound understanding of what was important and meaningful to them.

### Momentum points

While participants described how their *turning points* allowed agency through finding support, self-realization, and new meaning in life, they did not suggest that these consequences were the sole factors explaining the turn away from their *starting point*. This required consideration of *momentum points*, or events following the turning point that sustained or nurtured positive change. For participants whose turning points had occurred some years ago, *momentum points* contributed to an explicit acknowledgement of how participants were doing better now than at the starting point:

Pa8: This about being worried about “Do I have a job next week? […] Will I be able to pay for rent and everything else?” So, besides that, I feel incredible, in relation to how I felt many years ago.

Moreover, even when their current living conditions were not narrated as unambiguously positive, some participants’ identification of problems or difficulties included a description of them nonetheless having “a positive outlook on the future [I: Mm]. It feels fun and it feels good” (Pa3). The importance of *momentum points* in narrating growth after a turning point was particularly clear for Pa6, whose turning point had occurred just a few months before the interview. Pa6’s answer to how he was currently doing differed from what Pa8 mentioned above:

Pa6: I do have, eh, well an over-optimistic view of the future due to the new medication and such. But my situation right now is not good. [I: Mm] Well it is like this. [I: Mm] I am not where I want to be.

Therefore, in the following section, we describe the events that followed the *turning point* and were vital for participants’ improved mental health and well-being, grouped as *After, I will manage; After, I can move on*; and *After, I bring about change*.

### After, I will manage

After the *turning point*, participants started to define impending adversities as manageable; in other words, they reconstructed themselves as actors that could resolve rather than endure their problems. In some instances, agency was possible because the turning point was itself an alternative to their *starting point* context. Such was the case for Pa7, who said she used to self-harm to endure her father’s drinking habit, but discontinued self-harm when she could simply choose not to be present: “If I noticed that it would be one of those nights where dad would be drinking, then I could just go to my mum instead.” For others, the new context was in itself a valuable asset to them, making efforts to resolve adversity rather than endure it worthwhile. Pa4 said, for example, that she used to endure school-related anxiety and depression, but that at the *turning point* she could re-evaluate the relative importance of education; in other words, it was not worth returning to her *starting point* in exchange for a degree:

Pa4: Before it was like, it was [my education] that mattered. Nothing else. Eh, “I need to get through it, and that’s that.” Today, it is more like, if I can’t finish my education because I’m not feeling well, then it simply won’t happen either.

Pa4’s description of being able to appraise the negative effects of endurance was a recurrent theme in other stories as well. For example, Pa5 said he used to cope by “burying my head in the sand” but saw it within his capacity to resign from a management position when he realized its negative consequences for his health and well-being. For Pa4, Pa5, and others, the *turning point* became a new normal not only valuable in itself, but also a good foundation to build on as well as return to if they somehow were unsuccessful in managing their adversity. Pa1 explained this as not a matter of apprehending the lack of a resolution, but rather of dealing with things as they come: “Would I somehow end up in a situation where I don’t have a job anymore, then I am not afraid because I have so many things in my life […] that I can build my life on”.

However, participants also acknowledged situations that they were unable to manage themselves that put their *turning point* context at risk. In these instances, they explained how they relied on outside support:

Pa2: I got a lot of support from my parents when deciding that I wanted to quit [school] […] Otherwise I would probably have continued, and then I would have, well, felt even worse.

However, before the *turning point* where Pa2 expressed herself as being liberated from victimization, she—like many others—had expressed an unwillingness to seek social support. However, she and others narrated that, after the *turning point*, friends and relatives were pivotal to their management of difficulties by offering affirmation of their perspectives, or suggesting and supporting alternative action. Participants also described engaging in psychotherapy (Pa4, Pa10) or seeking help from public authorities (Pa8, Pa9) in difficult times after the *turning point*, which suggested that sensing agency after the *turning point* was important for utilizing interpersonal resources to manage and resolve adversity.

### After, I can move on

In instances where participants described that either the *starting point* or adverse events after the *turning point* were unsolvable, growth was nevertheless possible by moving on and constructing these experiences as moments of understanding one’s strengths and weaknesses. Moving on reinforced endurance as a temporary coping strategy when it had to be used at all, as Pa2 described after a difficult life event in adulthood: “When it happened, sometimes it was very difficult. But afterward, I processed it quickly afterward, [I: Mm] and I could let it go and view it as something positive.” Experiences of adversity could also be utilized to help others:

Pa8: I have hit rock bottom. And that has made me knowledgeable by like: “Okay, I’ve hit rock bottom, I know what that is like” and I try to help other people.

Viewing negative events as giving participants “something to bring along” (Pa4) also seemed to increase their self-perception as competent as well as their engagement with their environment. These consequences could even be framed as a future goal: “I have to work on allowing myself to fail [I: Mm] Because you have to allow yourself that” (Pa4). Consequently, while participants recognized that adversity was a necessary part of life, being able to reconstruct these events into moments of learning about themselves or how to help others made participants appreciate these experiences instead of constructing them setbacks.

### After, I bring about change

After the *turning point*, narrations of events related to life transitions, such as getting a job, finishing education, or settling into a domestic partnership, played a more significant role in the participants’ life stories than before. These events were reconstructed as affirmations of participants’ perceived ability to bring about change. In other words, instead of explaining them as chance events that “happen as you age” (Pa4), participants reinvented the events as accomplishments, prompting feelings of optimism and joy. For example, Pa8 narrated this as the delight he experienced in recollecting how he got his first job: “Then I got the call saying that I got the job, ‘Can you start on Monday? [I: Mm] YES! Oh, it was really, it was like the best birthday ever! Turning twenty-four and getting a job.” These events then became tangible milestones marking how participants had grown relative to their *starting point* context and how they, by their own merit, were building a life they considered to be worth living. These events reflected how they, as individuals, had the agency to bring about change.

## Discussion

The research questions that guided data generation and analysis in the current study concerned how young adults narrate life conditions and events occurring before, during, and after the discontinuation of NSSI, and how young adults construct agency in relation to these contexts and their understanding of who they are today. To explore these questions, we applied a narrative framework that included *turning points* (e.g., Clausen, 1998; Hareven & Masaoka, 1988; Pickles & Rutter, 1994; Wheaton & Gotlib, 1997) as well as the novel concepts of *starting points, no points*, and *momentum points*. Starting points aimed to capture the participants’ lived context prior to the turning point, which was characterized by narrations of enduring mental illness and repeated stressors through various strategies (e.g., NSSI). At the same time, attempts to change the situation by confiding in close relationships or starting therapy were constructed as no points. These events only changed the participants’ situation temporarily (if at all), often because the received support could not extend beyond the moment or was inadequate given what participants perceived themselves as enduring. Relative to this baseline, the turning point included events that contributed to participants feeling empowered to take on current challenges through a sense of belongingness, prioritizing one’s own wishes and needs after being liberated from a detrimental environment, and gaining perspective on which actions would support self-realization and which were unfavourable thereof. Therefore, while the characteristics of the turning point likely differed as a function of the fit between participants’ general dispositions and other life circumstances (Gilligan, 2009), the events shared a common theme of starting to reconstruct the self as someone who can manage their current problems. The importance of turning points in initiating NSSI discontinuation and psychological growth was previously described by Tofthagen et al. (2017), and in accordance with their work, we identified several subsequent factors and events essential for sustaining and nurturing positive psychological development. We called these momentum points, where participants narrated the management and resolution of adversity, how they moved on if this could not be the case, and how life transitions functioned as milestones to signify their achievements and motivated continued growth. While sensing agency was initially bound to the occurrence of the turning points, the momentum points pushed this sense of agency beyond the domains affected by a particular event. By recognizing that their perceived capacity to act after the turning point was greater than before, participants understood themselves as not only capable in the current moment, but also in movement towards a visualized future not previously achievable.

In accordance with earlier qualitative work (Donskoy & Stevens, 2013; Wadman et al., 2018), we found that adolescent NSSI was narrated in a low-agency context, where participants expressed that they could not otherwise cope with their experiences. This description of utilizing self-injury to endure a difficult situation has been previously described (Ekman & Söderberg, 2009; Long et al., 2015; Tofthagen et al., 2017; West et al., 2013), as has its co-occurrence with avoidant coping strategies such as cognitive disassociation and distraction (Andover et al., 2007). Therefore, self-injury was for participants in the current study not only made purposeful by the rationale of coping or regulating emotion as described by Edmondson et al. (2016), but also by contexts where other forms of coping (e.g., problem-solving, seeking social support) were possible but perceived as ineffective. Meanwhile, while the participants per Ammerman et al.’s (2017) definition quantitatively reported clinically significant levels of NSSI in adolescence, little significance was attributed to self-injury *per se*. NSSI was narratively situated as one method among others to manage contextual stressors, and not something intended for communicating distress to others (e.g., Holliday et al., 2018) or as causing distress in itself (e.g., Brown & Kimball, 2013). This implies that in the current study, NSSI was neither the source nor a symptom of their issues, meaning that discontinuing NSSI within this context was a change in endurance strategy rather than indicative of greater psychological well-being or improved life circumstances. Although overcoming an “urge” or “desire” to self-injure in triggering situations might represent an important milestone for some (Kelada et al., 2018; Kruzan & Whitlock, 2019; Lewis et al., 2019), our findings highlight that for others, psychological well-being or growth cannot be reduced to the cessation of NSSI (Warner & Spandler, 2012).

This finding suggested that a non-reductionist approach to understanding NSSI discontinuation and psychological growth aligns with the construction of well-being as more than the mere absence of self-destructive behaviours, mental illness, or detrimental living conditions (Ryff & Singer, 2003). In our study, sensing agency enabled participants to engage in more proactive means of managing distress (which is important for NSSI discontinuation, cf. Guerreiro et al., 2013) as well as made it possible for participants to start changing their current context into one where endurance was unnecessary. Other interview studies with related samples point in a similar direction, describing how individuals recognized the importance of self-initiative (Shaw, 2006) and personal determination (Sinclair & Green, 2005), found ways to increase their control over the environment (Buser et al., 2014; Kool et al., 2009), and realized their own intrinsic capacity to address current adversity (Long et al., 2015). However, our focus on sensing agency does not imply that the participants in this study could have made changes without environmental influences, as the strong association between gaining agency and a pivotal event highlighted its context-dependency (as also theorized by, e.g., Evans, 2007). Therefore, when applying a person-centred model of NSSI recovery as proposed by Lewis and Hasking (2021) that includes domains of self-efficacy, adversity management, and resilience, it is also necessary to consider the current lived context. Besides acknowledging that the discursive framework of recovery might not be relevant to everyone, an individual might want to (or is already taking action integral to) build resilience or thriving, but perceive themselves as impeded by difficult external situations that they cannot control. This could be a contributing factor to the ambivalence associated with discontinuing NSSI, as an individual might be hesitant to discontinue if it is beneficial to enduring or coping with events despite the acknowledged drawbacks (Gray et al., 2021).

Therefore, an essential part of supporting adolescents with self-injurious behaviours and/or mental illnesses might be to recognize the extent to which they perceive themselves as able to act in their current situation, rather than merely endure it. This might be particularly important because all participants who had sought counselling said it had been unhelpful prior to their turning point, and narratives about difficulties in engaging in healthcare are commonly reported by individuals who self-injure (Lindgren et al., 2018). Moreover, youth who are self-injuring also commonly report unique difficulties concerning ambivalence, honesty, engaging with difficulties beyond the therapy sessions, and decisions to keep up a façade and manage alone (Holliday et al., 2018). Furthermore, in accordance with Kelada et al. (2018), tensions and mistrust may be narrated when individuals perceive healthcare professionals to be focused more on their behaviours (e.g., self-injury) or symptoms (e.g., depressive symptoms) rather than on their context, or as our participants noted, they had a need to talk about what had happened to them. This has also been identified as a disadvantage of considering NSSI as a diagnosable entity in the Diagnostic and Statistical Manual of Mental Disorders (DSM-5) by other individuals with lived experience of NSSI (Lewis et al., 2017).

This suggest that for adolescents seeking outpatient care, a relational approach focusing on understanding the patients’ lived experience beyond their symptomology or fulfilled diagnostic criteria might facilitate more effective therapeutic relationships. This approach could also help build narrative coherence, which as Adler (2012) showed, longitudinally increases agency and improves mental health among individuals in psychotherapy. Moreover, Baerger and McAdams (1999) noted that narrative coherence was positively correlated with psychological well-being among American adults. Narrative coherence describes when episodes in a life story are linked to each other as well as to one’s sense of self (McAdams, 2001); in the context of the current study, this was represented by the momentum points. For example, growth-oriented experiences included the reconstruction of adversity into meaningful learning experiences and viewing life transitions as achievements, which contributed to participants’ narrating the self as competent and capable of initiating future change. While the cognitive capabilities that generate narrative coherence generally develop between adolescence and young adulthood (Habermas & Bluck, 2000), the incoherent and sometimes chaotic life narratives of adolescents (Hill & Dallos, 2012) and adults who self-injure (Chandler, 2014; Donskoy & Stevens, 2013) suggests this therapeutic approach may be particularly important for them. Moreover, when considering the reconstruction of lived experience, it is also important to recognize that positive turning points are retrospectively understood as such after the outcome is known (Gilligan, 2009). This suggests that helping young individuals identify current life changes that are expanding their sense of agency may not only increase their well-being temporarily, but also facilitate positive psychological growth in the longer run.

### Strengths and weaknesses

A strength of the current study is that we did not rely on retrospective reports of self-injury at the recruitment stage. Nor did we centre the interviews around self-injury in particular. These factors might explain the relatively low importance that participants attributed to the NSSI discontinuation process in comparison with earlier studies with community samples (e.g., Kruzan & Whitlock, 2019; Long et al., 2015). They also made it possible to include individuals who might not have been inclined to participate if NSSI had been the focus of the study (Potter & Hepburn, 2005). A particular highlight of the current study is that for some individuals who have previously injured themselves, the injury in itself is not necessarily a salient experience but rather one aspect among others in a difficult living situation. Since our participants did not construct NSSI as something negative in itself or narratively consequential, it is understandable that other topics such as distressing life events were perceived as more difficult to talk about. Nevertheless, none of our participants suggested that NSSI was an unanticipated or upsetting topic, and potential distress caused by sharing their experiences in general was temporary and/or limited relative to the positive aspects of doing so. This aligns with previous research suggesting that although NSSI is a stigmatized topic (Staniland et al., 2020), disclosing NSSI and other self-destructive behaviours in research is seldom associated with significant or long-term distress (Biddle et al., 2013).

Therefore, this study might include individuals with relevant experience who are otherwise difficult to reach while also posing minimal risk to their well-being, and thus can offer a more nuanced understanding of positive psychological development in adolescents who self-injure. However, although reporting NSSI over two data points in adolescence suggested that they had in fact engaged in that behaviour, and several interviews corroborated that the participants had self-injured in youth, we cannot confirm that the questionnaires adequately represented the extent of their adolescent self-injury. Nevertheless, we found several similarities between descriptions of self-injury and psychological growth in our study and previous qualitative work in community or clinical samples. Yet, it should be acknowledged that no participant described self-injury that could be lethal in terms of frequency or severity or could have life-long consequences beyond scarring. The narrated experiences of individuals who exhibited such behaviours in their past could be more stressful and chaotic than the experiences of those described in this study. Moreover, their self-injury could in itself have been constructed as an agentic expression, such as a means of re-establishing control in chaotic conditions (Donskoy & Stevens, 2013; Wadman et al., 2018) or reinstating the idea of an agentic self through harm to visible areas of the body (Csordas & Jenkins, 2018). Thus, while the results have limited generalizability to the entire population of individuals that self-injure while experiencing significant distress and trauma, the broad concepts are likely transferable to young adults who have experienced stress and mental illness in adolescence but currently perceive themselves as flourishing.

In relation to transferability, it is also important to acknowledge that our analysis was grounded in critical realism, meaning that we assumed that the participants’ narratives reflected events that actually occurred. People do tend to be subjectively and objectively truthful in their narrative accounts (Andrews, 2020), and the interviewer’s experience as a licenced clinical psychologist might have afforded trust and comfort in the interview situation. However, as acknowledged by researchers such as Mishler (1986), narratives are also joint construction between speaker and listener, and what or how something is told varies with the conditions of the interview. As turning points are viewed as such from one’s current context (Gilligan, 2009), not only might participants describe the event that turned their life around differently at another time, but the event could be reconstructed as not being a turning point at all. This means that the data generated and analysed in this study do not describe positive psychological development insofar as it does participants’ current view of their growth. Nevertheless, these views are meaningful descriptions of how young adults perceive themselves as flourishing despite prior adversities, and illustrate the life circumstances that can be important in the development of psychological well-being for adolescents who struggle regardless of whether they self-injure or not.

Finally, while ten participants in the current study narrated a turning point, one participant did not. In congruence with our interpretation that gaining agency was a necessary condition of narrating momentum points, this participant also did not recount the experience of any momentum points. Our reading of the interview suggested that this participant’s well-being was lower than that of the rest of the sample, even though she had discontinued NSSI and was living under different conditions from those in her adolescence. This reading is also supported by previous research that associated narrations of positive turning points and agency with higher well-being and better mental health (Adler, 2012; Bauer & McAdams, 2004; Tavernier & Willoughby, 2012). However, while this contrasting narrative provides some support for the results, one case is not sufficient for providing a thick description of the narrative implications of not having experienced an agentic shift. Therefore, we suggest that future research explore this further by studying the factors impeding improved well-being after discontinuing NSSI, particularly since we cannot exclude that the narrative coherence suggested by momentum points could be the result of lingual and cognitive development (Habermas & Bluck, 2000). Moreover, the majority of the starting points in this study were narrated as occurring in adolescence, during which agency is bounded by social structures in such areas as education and family (Evans, 2007), which may change after reaching the age of majority. Therefore, applying our narrative framework of no points, turning points, and momentum points to narratives from individuals older than 25 but who do not thrive until even later in life might also be promising in understanding the development of eudaimonic well-being in association with perceived agency and life events.

## Conclusions

Through a narrative analysis of interviews of young adults reporting discontinuation of NSSI in adolescence, we found that before an event that constituted an agentic shift in the narrative, participants described themselves as constrained within their context. However, as pivotal as the turning point was for narrating agency and subsequent psychological growth, subsequent events that nurtured and sustained this development were equally important for well-being. Besides outlining how the discontinuation of NSSI is only indicative of psychological growth within life conditions that facilitate agency, our results also tentatively illustrate how life events play a pivotal role in psychological growth for individuals who discontinue self-injury. Moreover, to support adolescents and young adults who self-injure or have mental health problems, focusing solely on assessing their behaviours or treating their symptoms might unintentionally disregard whether an individual regards themselves as being able to engage with the support given.
